# BCG-booster vaccination with HSP90-ESAT-6-HspX-RipA multivalent subunit vaccine confers durable protection against hypervirulent Mtb in mice

**DOI:** 10.1038/s41541-024-00847-7

**Published:** 2024-03-08

**Authors:** Kee Woong Kwon, Han-Gyu Choi, Kwang Sung Kim, Shin Ae Park, Hwa-Jung Kim, Sung Jae Shin

**Affiliations:** 1 Department of Microbiology, Graduate School of Medical Science, Brain Korea 21 Project, Yonsei University College of Medicine, Seoul, 03722 South Korea; 2 Department of Microbiology, College of Medicine, Gyeongsang National University, Jinju, 52727 South Korea; 3 Department of Microbiology, and Medical Science, College of Medicine, Chungnam National University, Daejeon, 35015 South Korea; 4 R&D Center, EyeGene Inc., Goyang, 10551 South Korea; 5 Institute for Immunology and Immunological Disease, Yonsei University College of Medicine, Seoul, 03722 South Korea

**Keywords:** Protein vaccines, Adjuvants

## Abstract

The quest for effective and enhanced multiantigenic tuberculosis (TB) subunit vaccine necessitates the induction of a protective pathogen-specific immune response while circumventing detrimental inflammation within the lung milieu. In line with this goal, we engineered a modified iteration of the quadrivalent vaccine, namely HSP90-ESAT-6-HspX-RipA (HEHR), which was coupled with the TLR4 adjuvant, CIA09A. The ensuing formulation was subjected to comprehensive assessment to gauge its protective efficacy against the hypervirulent *Mycobacterium tuberculosis* (Mtb) Haarlem clinical strain M2, following a BCG-prime boost regimen. Regardless of vaccination route, both intramuscular and subcutaneous administration with the HEHR vaccine exhibited remarkable protective efficacy in significantly reducing the Mtb bacterial burden and pulmonary inflammation. This underscores its notably superior protective potential compared to the BCG vaccine alone or a former prototype, the HSP90-E6 subunit vaccine. In addition, this superior protective efficacy was confirmed when testing a tag-free version of the HEHR vaccine. Furthermore, the protective immune determinant, represented by durable antigen-specific CD4^+^IFN-γ^+^IL-17A^+^ T-cells expressing a CXCR3^+^KLRG1^-^ cell surface phenotype in the lung, was robustly induced in HEHR-boosted mice at 12 weeks post-challenge. Collectively, our data suggest that the BCG-prime HEHR boost vaccine regimen conferred improved and long-term protection against hypervirulent Mtb strain with robust antigen-specific Th1/Th17 responses.

## Introduction

Tuberculosis (TB) stands as the primary cause of mortality attributed to a single infectious agent, *Mycobacterium tuberculosis* (Mtb). According to the Global TB Report 2022, approximately 10.4 million new TB cases emerged^1^. Notably, nearly a quarter of the global population in Asia and Africa harbors latent Mtb infection, with ~5–10% of these individuals possessing an elevated risk of transitioning to full-blown disease over their lifetimes^1^. In the aftermath of the World Health Organization’s (WHO) declaration of COVID-19 as a pandemic, the COVID-19 pandemic has adversely influenced TB patient care, leading to increased diagnostic delays, reduced hospital admissions, and the disruption of treatment services^2,3^. Vaccines are an effective approach for managing infectious disease outbreaks and mitigating pandemic and epidemic risks^4^. In this regard, the sole TB vaccine approved for clinical use remains Bacille Calmette-Guérin (BCG), a live attenuated *Mycobacterium bovis* strain introduced in 1921^5^. Although BCG administration at birth significantly curtails the incidence of severe miliary and meningeal TB in infants and children, its efficacy against pulmonary TB is comparatively limited^6^. A more potent and secure alternative to BCG, or even BCG-prime boosting, is urgently warranted.

Among several TB vaccine types, a protein-based subunit vaccine has been especially regarded as highly appealing due to its safety profile^7^. Furthermore, this type of vaccine offers the advantage of flexibility, allowing for various approaches to address the limitations of the existing BCG vaccine. This can be achieved by either enhancing BCG-primed immune response, incorporating Ags present in BCG, or utilizing Ags not expressed and/or secreted by BCG^8–11^. TB vaccines under clinical trials predominantly focus on prophylactic applications, targeting antigens (Ags) primarily expressed during the initial infection phase. However, these early Ags undergo substantial downregulation during the subsequent dormant state of the bacteria^12^. Given that the majority of TB patients experience latent infection preceding active disease onset, Ags associated with latency and immunologically discernible during this phase assume importance in TB vaccine development^13^. Hence, leading TB subunit vaccine candidates, such as ID93/GLA-SE and H56:IC31, target multiple stages of Mtb infections, particularly by utilizing latency-associated Ags, and are presently undergoing clinical phase 2 trials^10,14,15^. Therefore, the selection of suitable Ags for the development of an enhanced TB subunit vaccine is crucial.

However, using Mtb Ag alone is insufficient to generate a strong immune response; activating innate immune cells and providing co-stimulatory signals through adjuvants is essential for effective Ag-specific T-cell response. Like AS01_E_, which is also an adjuvant for a leading TB vaccine^8,16^, CIA09A is an adjuvant system composed of 1,2-dioleoyl-3-trimethylammonium-propane (DOTAP)-based cationic liposomal formulation containing a mixture of TLR4 ligand de-*O*-acylated lipooligosaccharide (dLOS) and saponin fraction QS-21. CIA09A amplifies both antibody and cell-mediated immune responses to various antigens, including recombinant TB antigens, inactivated Japanese encephalitis vaccine (JEV), and recombinant varicella-zoster virus (VZV) glycoprotein E (gE) antigen, while notably excelling in inducing a Th1-biased response characterized by IFN-γ cytokine production^17–19^. Investigation into the mechanism of CIA09A’s action using the VZV gE antigen model has highlighted its role in augmenting antigen stability, aiding antigen uptake at the injection site, attracting immune cells, facilitating antigen delivery to lymph nodes, and aiding APC-mediated antigen presentation to T-cells^20^. This underscores the significance of understanding how vaccination routes impact immune responses. The choice of vaccination route significantly affects vaccine effectiveness by influencing immune cell priming and subsequent local and systemic immune responses. Different routes, such as intramuscular and mucosal administration, have shown varying outcomes in TB vaccine research, with the potential for inducing protective immune responses^21–23^. These insights emphasize the need to explore optimal routes for vaccine delivery to harness their full potential in eliciting protective immunity.

The heterogeneity in phenotypic, genotypic, and pathogenic attributes among various Mtb strains should not be underestimated, as these elements can significantly influence vaccine efficacy^24,25^. Different Mtb strains exhibit varying levels of virulence^24^, with Mtb lineage 4, Euro-American strain families, is considered a modern lineage consisting of lineages 2 to 4. These modern lineages are the primary cause of globally distributed TB epidemics and, consequently, most of the TB disease burden. Among these, Mtb lineage 4 has the broadest range, encompassing regions across the Americas, Asia, Africa, and Europe^26,27^. Therefore, it has been suggested that the assessment of vaccine effectiveness against prevailing clinical strains of Mtb may be taken into account in the preclinical stage of vaccine development^28,29^. In addition, most preclinical studies evaluating the efficacy of new TB vaccine candidates in mice specifically concentrate on measuring Mtb replication in the lungs only at 4 weeks post-infection, with limited exploration of extended protection^30^. In this regard, it is notable that in C57BL/6 mice, the BCG Pasteur vaccine significantly reduced lung CFU for 9 distinct Mtb strains at 4 weeks post-infection, but this reduction gradually diminished in later stages^24^. Moreover, in a previous study, we observed that the protective efficacy of both BCG Danish and Pasteur vaccines was insufficient against chronic infection caused by Mtb clinical strains^31^. This indicates that ensuring long-term protection against Mtb clinical strains is essential for a TB vaccine.

Previously, we evaluated the vaccine efficacy of HSP90-E6/CIA05 + DDA against Mtb clinical strain HN878^32^. In the current study, we have constructed the advanced subunit vaccine, HEHR, beyond HSP90-E6 by incorporating heat-shock protein X (HspX) and resuscitation-promoting factor interacting protein (RipA), which have been reported as TB vaccine antigens, including our ongoing research^33,34^. Subsequently, the HEHR formulation in the CIA09A adjuvant, currently being considered for various clinical trials, was evaluated as a potential BCG prime booster candidate against chronic infection caused by the Mtb clinical strain M2, where significant BCG-derived protection was not achieved^31^. Boosting BCG with HEHR/CIA09A via both homologous and heterologous routes provided superior and long-lasting protection accompanied by robust Th1/Th17 responses, positioning it as an advanced vaccine candidate compared to HSP90-E6/CIA09A. Moreover, a tag-free version of HEHR significantly improved BCG-primed efficacy by ameliorating lung inflammation and mediating bacterial reduction. Our results provide the rationale for further exploration and more feasible validation of HEHR/CIA09A as a BCG-prime booster vaccine.

## Results

### Screening of multivalent fusion antigens and boosting BCG with HEHR/CIA09A induces antigen-specific multifunctional CD4^+^ T-cell responses

**Fig. 1:**
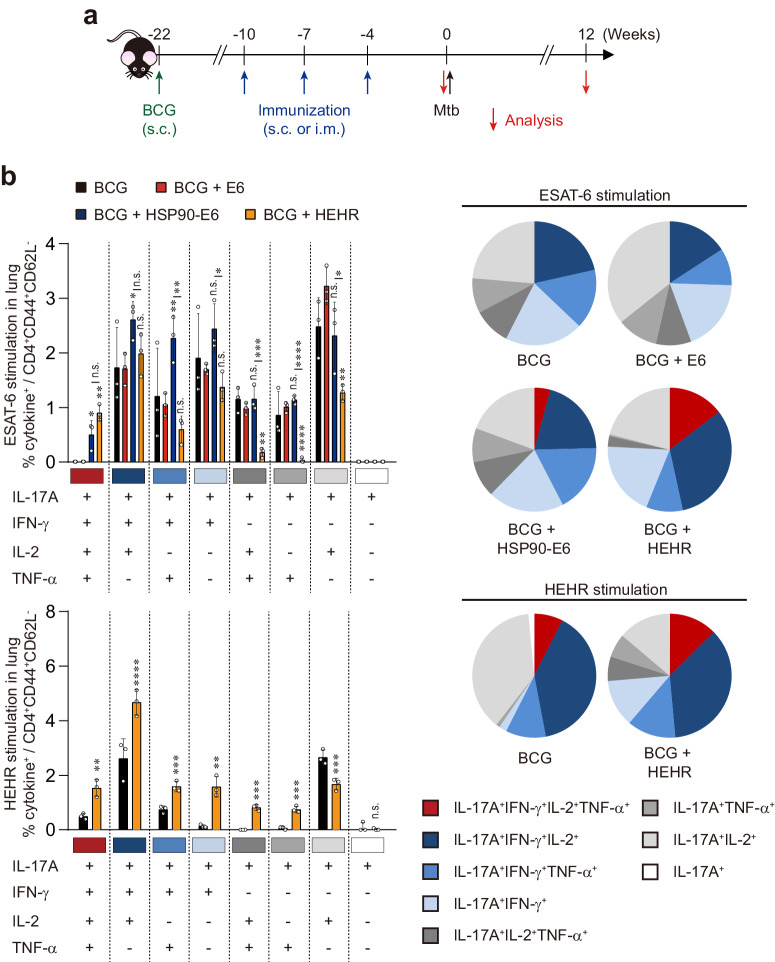
Analysis of Ag-specific multifunctional T-cells in BCG + HEHR/CIA09A-immunized mice. **a** Schematic diagram in each panel shows the detailed vaccination, boosting, and infection schedule. Mice (*n* = 3 per group) were immunized by BCG injection 12 weeks before subunit vaccination. Three intramuscular or subcutaneous injections of E6/CIA09A, HSP90-E6/CIA09A, and HEHR/CIA09A were administered (blue arrows) before the Mtb M2 aerosol challenge (black arrow). Immunological analysis was conducted before and after the Mtb infection (red arrow). Bacterial counts and histopathology in each group were determined at the indicated time points after Mtb infection. **b** Mice were immunized and euthanized as described in the methods section. Four weeks after the last immunization, the mice were sacrificed, and their lung cells collected from the mice were treated with ESAT-6 (2 μg/ml) or HEHR (2 μg/ml) at 37 °C for 12 h in the presence of GolgiStop. Upon stimulation with ESAT-6 or HEHR, cell counts of Ag-specific, multifunctional CD4^+^CD44^+^CD62L^-^ T-cells producing IFN-γ, IL-17A, and/or TNF-α and IL-2 in the lung cells from each immunized group were determined by flow cytometry. Data were expressed as the mean ± SD for three mice from each group. Data were analyzed by one-way ANOVA with post hoc Tukey test. n.s. not significant, *p < 0.05, **p < 0.01, ***p < 0.001, and ****p < 0.0001. The level of significance determined between BCG- and BCG + HEHR-immunized mice; ns: not significant, **p < 0.01, ***p < 0.001, and ****p < 0.0001.

It is important to note that the order of antigen placement in fusion protein creation can influence the outcome^35^. As a result, we constructed four recombinant fusion proteins, namely HSP90-ESAT6-RipA (HER), HSP90-ESAT6-HspX (HEH), HSP90-ESAT6-RipA-HspX (HERH), and HSP90-ESAT6-HspX-RipA (HEHR), all anchored around HSP90-ESAT6 (Supplementary Fig. 1a; all the uncropped and unprocessed gel images are provided in Supplementary Fig. 7). Prior to commencing the study, we assessed the cytotoxicity of the purified recombinant fusion proteins (Supplementary Fig. 1b), and none of tested fusion proteins induced cytotoxic effect on dendritic cells (DCs). Next, considering that DCs play an important role as primary mediators between innate and acquired immunity by processing and presenting antigens to induce T-cell immunity, we thus screened whether the four recombinant fusion proteins affect the maturation status of DCs. Stimulation of all fusion proteins, except for HER, induced elevated expression of CD86 and MHC-II, and increased production of inflammatory cytokines compared to non-stimulated DCs (Supplementary Fig. 2a, b). Transitioning to the interaction between activated DCs and naïve CD4^+^ T-cells, critical for T-cell activation, we initiated a T-cell proliferation assay using ovalbumin (OVA)-specific CD4^+^ T-cells from OT-II TCR transgenic mice. As expected, HEH, HERH, and HEHR-treated DCs, pulsed with OVA_323-339_, led to greater T-cell proliferation compared to OVA-pulsed or LPS-treated DCs (Supplementary Fig. 3a), along with the increased production of Th1/Th17-biased cytokines (Supplementary Fig. 3b). To explore further, we examined whether T-cells activated by fusion proteins-treated DCs could inhibit mycobacterial growth in bone marrow-derived macrophages (BMDMs). Notably, T-cells from BCG-vaccinated mice, subsequently activated by HEHR-treated DCs, only displayed remarkable bacterial growth control within BMDMs, by reducing the initial infection loads (Supplementary Fig. 3c). Collectively, among the four recombinant fusion proteins, HEHR displayed optimal DC activation potential, increasing the production of Th1/Th17-related cytokines and enhancing control over mycobacterial growth. Subsequently, we chose the HEHR fusion protein to proceed with evaluating protective potential. The use of a homologous route for prime-boost vaccinations, where both vaccinations target the same draining lymph nodes (LNs), is more effective in providing a boost compared to heterologous routes, which involve vaccinations targeting different or distant LNs^36^. Thus, mice were subcutaneously primed with BCG and subsequently boosted via the same route with HEHR formulated in CIA09A adjuvant (Fig. 1a). Based on our prior findings indicating the efficacy of Th1/Th17 in Mtb control^32^, the multifunctional T-cells, characterized by IL-17A and IFN-γ production, were analyzed from CD4^+^ T-cells collected from the lungs using multi-color flow cytometry (following the gating strategy outlined in Supplementary Fig. 4). BCG-primed HEHR/CIA09A boosted immunization prompted the expansion of Ag-specific CD4^+^CD44^+^CD62L^-^ multifunctional T-cells (IFN-γ^+^IL-17A^+^TNF-α^+^IL-2^+^ and IFN-γ^+^IL-17A^+^IL-2^+^ cells) to an extent comparable to HSP90-E6/CIA09A boosted immunization upon re-stimulation with ESAT-6. Furthermore, re-stimulation with HEHR led to an even greater expansion of Ag-specific CD4^+^CD44^+^CD62L^-^ multifunctional T-cells in the BCG-primed HEHR/CIA09A boosted group compared to those of BCG-primed only mice (Fig. 1b). In line with this, upon ex vivo stimulation of lung cells with ESAT-6 or HEHR, significantly elevated secretion of T-cell polyfunctionality-related cytokines was observed in HEHR/CIA09A-boosted mice compared to those of HSP90-E6/CIA09A boosted mice (Supplementary Fig. 5). These findings underscore that BCG-primed HEHR/CIA09A boosted regimen elicits HEHR-specific Th1/Th17 responses, characterized by Ag-specific polyfunctional properties within the lung.

### Protective efficacy of BCG prime HEHR/CIA09A boosting against the hypervirulent M2 strain

**Fig. 2:**
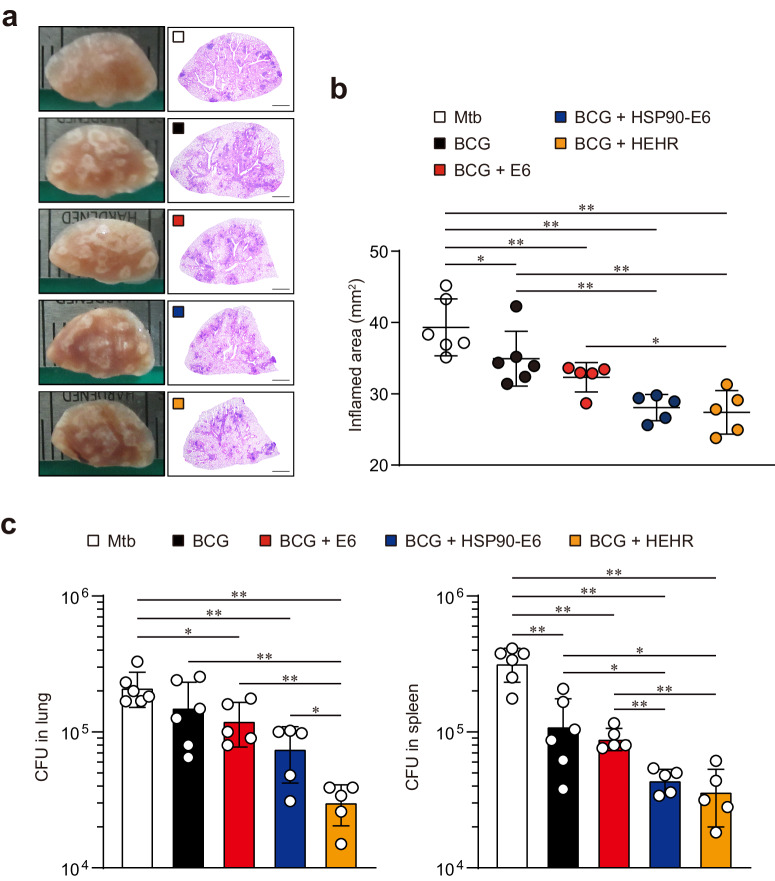
Intramuscular administration of HSP90-E6/CIA09A and HEHR/CIA09A booster vaccination improves BCG-primed protection against hypervirulent Mtb M2. **a** H&E staining of superior lobes of the right lung of each immunized mouse (*n* = 5 or 6) at 12 weeks after Mtb M2 infection (scale bars = 2.0 mm). **b** Inflamed lesion size in the lungs. **c** CFUs in the lungs and spleen in all treatment groups at 12 weeks post-infection, determined by counting the viable bacteria. The graph shows the mean ± SD. Mann–Whitney rank tests were used to compare groups. *n.s*. not significant, *p < 0.05 and **p < 0.01.

**Fig. 3:**
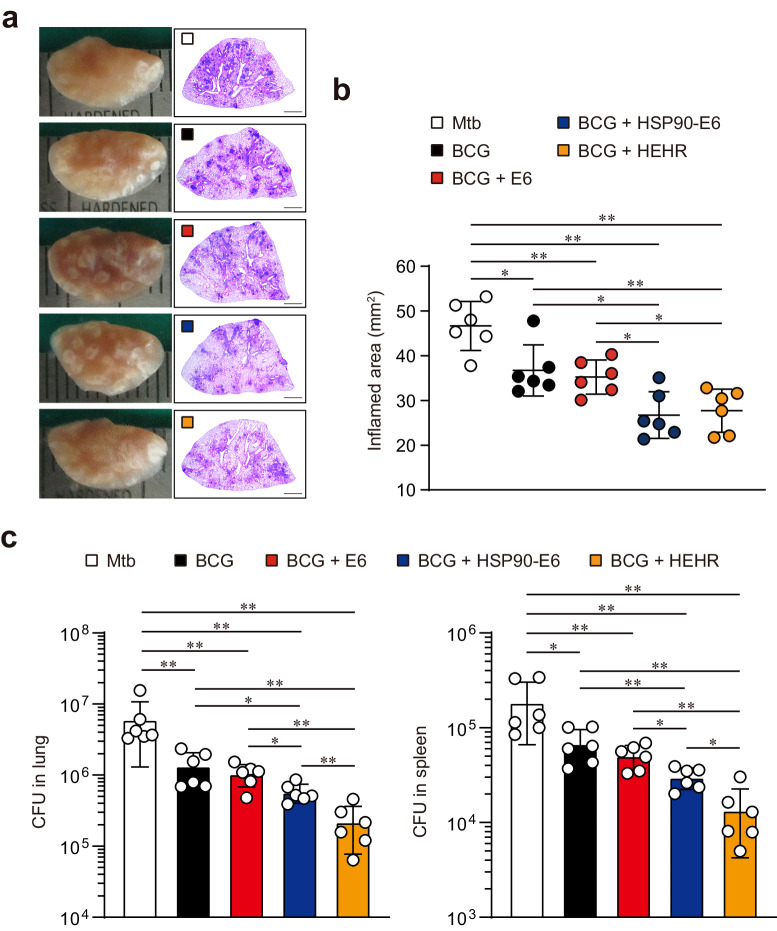
Subcutaneous administration of HSP90-E6/CIA09A and HEHR/CIA09A booster vaccination improves BCG-primed protection against hypervirulent Mtb M2. **a** H&E staining of superior lobes of the right lung of each immunized mouse (*n* = 6) at 12 weeks after Mtb M2 infection (scale bars = 2.0 mm). **b** Inflamed lesion size in the lungs. **c** CFUs in the lungs and spleen in all treatment groups at 12 weeks post-infection, determined by counting the viable bacteria. The graph shows the mean ± SD. Mann–Whitney rank tests were used to compare groups. *n.s*. not significant, *p < 0.05 and **p < 0.01.

In previous study, our group reported that BCG-primed boost vaccination using HSP90-E6/CIA05 via a heterologous route resulted in a significant augmentation of CD4^+^ T-cell responses. These responses were particularly characterized by an enrichment of Th1/Th17 polarized cells, which exhibited a correlation with protection against infection by the highly virulent Mtb HN878 strain^32^. To explore further, we aimed to determine whether this effect could be amplified using the more advanced HEHR vaccine compared to HSP90-E6. Thus, as an independent experimental setting, BCG-primed HEHR/CIA09A intramuscularly boosted mice were challenged with Mtb M2 strain (Fig. 1a). In line with observations in BCG-primed HEHR/CIA09A subcutaneously boosted mice (Supplementary Fig. 5), upon ex vivo stimulation of lung cells with ESAT-6 or HEHR, elevated production of T-cell polyfunctionality-associated cytokines was also observed in HEHR/CIA09A-boosted mice (Supplementary Fig. 6). At 12 weeks post-infection, HEHR/CIA09A-boosted mice only conferred significant amelioration in lung inflammation compared to ESAT-6/CIA09A-boosted group (Fig. 2a, b). HSP90-E6/CIA09A-boosted vaccination exhibited partial protection against the Mtb lineage 4 M2 strain, similar to observations during Mtb lineage 2 HN878 infection^32^. This effect was characterized by a significant reduction in bacteria burden, notably in the spleen, in comparison to that of mice boosted with ESAT6/CIA09A. As observed in the HSP90-E6/CIA09A-boosted group, HEHR/CIA09A-boosted mice also mediated improved protection in the spleen. Moreover, HEHR/CIA09A-boosted vaccination mediated improved protection by significantly reducing bacterial burden compared to those of both ESAT-6/CIA09A- and HSP90-E6/CIA09A-boosted mice (Fig. 2c). These results indicate that boosting BCG with HEHR/CIA09A via intramuscular route provided significant protection against Mtb M2 infection, notably exhibiting a superior level of protection especially in the lung. Furthermore, in line with Fig. 1, prime-boost vaccinated mice with homologous routes were challenged with the Mtb M2 strain. Contrary to the effects of boosting via a heterologous route, both subcutaneously HSP90-E6/CIA09A- and HEHR/CIA09A-boosted mice demonstrated a notable reduction in lung inflammation compared to those that received BCG prime only or were boosted with ESAT6/CIA09A (Fig. 3a, b). Importantly, while no significant difference was noted in lung inflammation between these two groups, the HEHR/CIA09A-boosted group exhibited significantly lower bacterial burden in both the lung and spleen compared to the HSP90-E6/CIA09A-boosted mice (Fig. 3b, c). Collectively, the HEHR/CIA09A vaccine candidate exhibited significant protective potential as a BCG booster, irrespective of the boosting route, by surpassing the long-term efficacy against Mtb M2 infection observed in the HSP90/CIA09A-boosted regimen.

### Analysis of the Ag-specific immune responses in the lungs of BCG prime HEHR/CIA09A boosting after challenge with Mtb M2

**Fig. 4:**
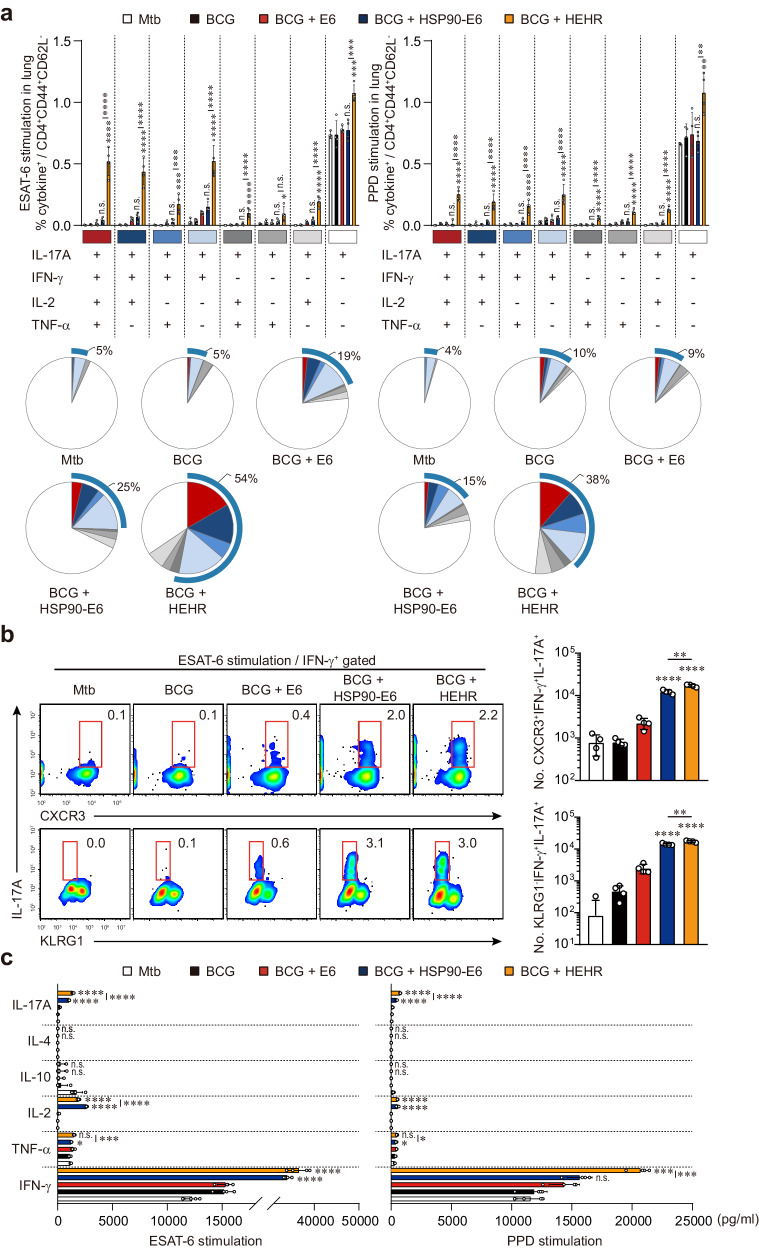
Antigen-specific multifunctional T-cell subsets and cytokine production after challenge with Mtb M2. **a** Mice of each group were sacrificed at 12 weeks post-infection, and lung cells obtained from the mice were treated with ESAT-6 (2 μg/ml) or PPD (2 μg/ml) at 37 °C for 12 h in the presence of GolgiStop. Upon stimulation with the ESAT-6 or PPD, cell counts of Ag-specific, multifunctional CD4^+^CD44^+^CD62L^-^ T-cells producing IFN-γ, IL-17A, and/or TNF-α and IL-2 in the spleen and lung cells in all treatment groups were determined by flow cytometry. Blue arc denotes the percentage of cytokine-positive T-cells (IL-17A^+^IFN-γ^+^TNF-α^+^IL-2^+^-, IL-17A^+^IFN-γ^+^IL-2^+^-, IL-17A^+^IFN-γ^+^TNF-α^+^-, and IL-17A^+^IFN-γ^+^-CD4^+^CD44^+^ T-cells). Data from one representative experiment are presented as the mean ± SD from pooled samples (*n* = 4) from each group (*n* = 6). Data were analyzed by one-way ANOVA with post hoc Tukey test. *n.s*. not significant, *p < 0.05, **p < 0.01, ***p < 0.001, and ****p < 0.0001. **b** Mice of each group were sacrificed at 12 weeks post-infection, and lung cells obtained from the mice were treated with ESAT-6 (2 μg/ml) at 37 °C for 12 h. Upon stimulation with the ESAT-6, cell counts of Ag-specific, multifunctional CD4^+^CD44^+^IFN-γ^+^IL-17A^+^ and CXCR3^+^ or KLRG1^-^ in the lung cells in all treatment groups were determined by flow cytometry. Data from one representative experiment are presented as the mean ± SD from pooled samples (*n* = 4) from each group (*n* = 6). ****p < 0.0001 compared to BCG-immunized mice. **p < 0.01 between BCG + HSP90-E6- and BCG + HEHR-immunized mice. **c** Levels of IFN-γ, TNF-α, IL-2, IL-10, IL-4, and IL-17A secreted by lung and spleen cells in all treatment groups in response to ESAT-6 (2 μg/ml) or PPD (2 μg/ml) stimulation as detected by ELISA. Data from one representative experiment are presented as the mean ± SD from pooled samples (*n* = 4) from each group (*n* = 6). *n.s*. not significant, *p < 0.05, ***p < 0.001, and ****p < 0.0001 compared to BCG-immunized mice. *p < 0.05, ***p < 0.001, and ****p < 0.0001 between BCG + ESAT-6- and BCG + HSP90-E6-immunized mice.

We then explored whether the magnitude and quality of polyfunctional responses, characterized by IFN-γ and IL-17A production, could be sustained or augmented upon Ag re-stimulation. At 12 weeks post Mtb M2 infection, lung cells were ex vivo stimulated with ESAT-6 and PPD, and Ag-specific CD4^+^ T-cells were then analyzed using multi-color flow cytometry. The frequency of ESAT-6-specific CD4^+^CD44^+^CD62L^-^ multifunctional T-cells secreting IL-17A, IFN-γ, IL-2, and/or TNF-α in the lung of mice in the BCG-primed HEHR/CIA09A boosted group was maintained at 12 weeks after challenge with Mtb M2. Under PPD stimulation, the immune responses primed by BCG appeared to be enhanced in the lung through HEHR/CIA09A vaccination, as indicated by the increase of PPD-specific multifunctional T-cells (Fig. 4a). Recent studies have highlighted the significance of T-cell properties beyond multi-functionality, emphasizing the acquisition of an effector phenotype, including lung-homing associated markers like CXCR3 and KLRG1, as crucial for controlling Mtb^37–39^. In HEHR/CIA09A-boosted mice, a remarkable elevation in Ag-specific T-cells co-producing IFN-γ and IL-17A was noted, accompanied by increased CXCR3 expression and decreased KLRG1 expression in the lung compared to those observed in HSP90-E6 boosted mice (Fig. 4b). Next, we investigated cytokine profiles in the lungs upon ex vivo stimulation of PPD or ESAT-6 to determine whether Th1/Th17-mediated responses were maintained. Consistent with the findings in flow cytometric analysis, there was a remarkable increase in the production of Th1/Th17-associated cytokines, such as IFN-γ, TNF-α, IL-2, and IL-17A, while Th2 cytokines, namely IL-4 and IL-10, remained unchanged in the lung of BCG-primed HEHR/CIA09A boosted mice upon both ESAT-6 and PPD ex vivo stimulation. Additionally, the production of IL-17A following ESAT-6 stimulation and the production of IFN-γ and IL-17A after PPD stimulation were further heightened in HEHR/CIA09A boosted mice compared to those in HSP90 + E6/CIA09A boosted mice (Fig. 4c).

### Confirmation of protective efficacy of BCG primed tag-free HEHR/CIA09A boosted regimen against Mtb M2 strain

**Fig. 5:**
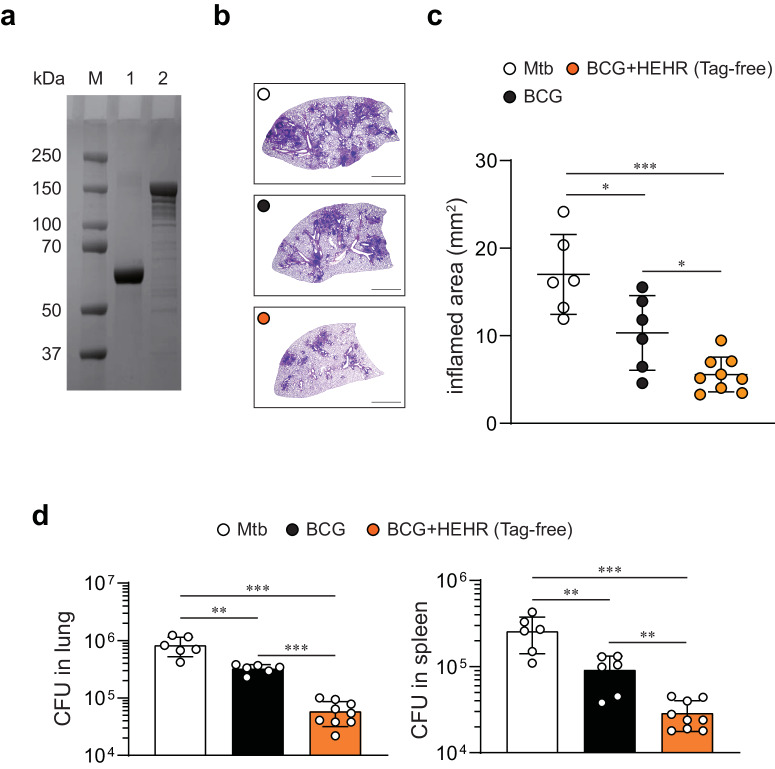
Subcutaneous administration of tag-free HEHR/CIA09A booster vaccination improves BCG-primed protection against hypervirulent Mtb M2. **a** Purified recombinant tag-free HEHR protein was analyzed by SDS-PAGE. M: Protein Marker, Lane 1: BSA, Lane 2: tag-free HEHR. The gel was derived from the same experiment and was processed in parallel. **b** H&E staining of superior lobes of the right lung of each immunized mouse (*n* = 6 or 9) at 12 weeks after Mtb M2 infection (scale bars = 2.0 mm). **c** Inflamed lesion size in the lungs. **d** CFUs in the lungs and spleen in all treatment groups at 12 weeks post-infection, determined by counting the viable bacteria. The graph shows the mean ± SD. Mann–Whitney rank tests were used to compare groups. **p < 0.01 and ***p < 0.001.

Considering the potential variation in immunogenicity and efficacy attributed to the presence or absence of a His-tag^40^, we adopted a strategy to produce the HEHR fusion protein without a His-tag, intending to progress toward future clinical trials (Fig. 5a; all the uncropped and unprocessed gel images are provided in Supplementary Fig. 7). Then, we further evaluated whether tag-free HEHR adjuvanted in CIA09A can also confer long-term protection as BCG booster against Mtb M2 infection. Notably, BCG-primed tag-free HEHR/CIA09A-boosted mice via homologous route showed a significant reduction in pulmonary inflammation and bacterial burden compared to mice solely primed with BCG (Fig. 5b–d). In summary, our findings supported the potential of the more advanced HEHR/CIA09A vaccine as a TB vaccine candidate, further confirmed by the enhanced protection observed in BCG-primed tag-free HEHR/CIA09A boosted mice.

## Discussion

In this study, we combined our previous fusion proteins, HSP90-E6 with HspX-RipA, to create a multi-stage fusion vaccine Ag target named HEHR. Our findings reveal that HEHR exhibits higher immunogenicity and superior protective efficacy against Mtb M2 infection compared to a previous vaccine candidate, HSP90-E6. Boosting BCG with HEHR/CIA09A through intramuscular and subcutaneous injections were both effective in safeguarding against chronic infection with Mtb M2 accompanied by significant induction of Ag-specific multifunctional T-cells displaying effector phenotypes. In addition, enhancing BCG-priming efficacy mediated by HEHR/CIA09A boosted vaccination was further confirmed by employing a tag-free version of HEHR. Overall, our findings emphasize the potential of the HEHR/CIA09A vaccine due to its ability to provide long-lasting protection against Mtb lineage 4, which spans across the broadest range among modern Mtb lineages. These results suggest the importance of further investigation for feasible validation as a BCG booster candidate.

When Mtb infects the lungs, a coexistence of rapidly growing and nonreplicating dormant forms is observed. These bacterial populations exist along a continuum with varying metabolic states, capable of interconversion^41^. In the primary phase, rapid growth predominates, alongside a smaller population of non-growing bacteria. As the infection progresses, the number of growing bacteria diminishes while the latent population increases. These dynamic underscores the need to include antigens from different growth stages when constructing multi-stage TB subunit vaccines. This strategy ensures comprehensive immune protection against bacteria in diverse metabolic states^42,43^. In addition to ID93 and H56 vaccines which at the forefront in clinical trials^10,14,15^, for instance, the fusion protein LT69 containing HspX displayed robust immunogenicity and notable protection against Mtb^34^. Another fusion protein LT70 containing Rv2626c, demonstrated vaccine potential as BCG prime booster by conferring long-term protection^44^. As a single Ag, Rv1733c, latent-related Ag, demonstrated a significant reduction in bacterial load in Mtb-challenged mice when administered as synthetic peptides^45^. Our previous study also suggested the vaccine potential of latency-related Ag, RipA, against Mtb clinical strains as a single Ag^33^. Motivated by this, we constructed an advanced TB subunit vaccine based on the previous version, HSP90-E6, by combining HspX and RipA. Considering the influence of antigen placement order in a fusion protein on efficacy outcomes^35^, we finalized HEHR based on the antigen’s capacity to induce protective Th1 immune responses through dendritic cell activation, a methodology consistently employed in our identification of TB vaccine antigens^33,46–50^. Notably, it was observed that HEHR more efficiently induced the production of IL-1β, IL-12p70, and IL-23p19 than HERH did, although they have the same components. This may be attributed to structural changes as variations in protein structure can lead to diverse capacities for Ags to interact with cells and receptors crucial for initiating the immune response^51^. Additionally, it was reported that a single amino acid mutation can induce a structural change, resulting in alterations in the response of innate immune cells^52,53^. Although we did not directly analyze in the current study, it deserves further consideration, given that structure-based Ag design is one of the crucial strategies for the development of effective subunit Ags^54^. Building on these insights, subsequently, HEHR was evaluated as a BCG booster formulated in CIA09A, currently under consideration for various clinical trials.

The administration route of vaccination can profoundly influence vaccine immunogenicity and efficacy, as the localization of the vaccine critically determines the efficient priming of immune cells, leading to optimal local and systemic immune responses^55^. For instance, intramuscular immunizations of mice with live-attenuated or TB subunit vaccines induced more rapid antibody production than the subcutaneous route^56–58^. Additionally, the mucosal route, including intratracheal or intranasal vaccination, has piqued interest in TB vaccine research. Pulmonary delivery methods such as BCG^21,59^, live recombinant viruses^60–62^, and protein/adjuvant combinations^23,63,64^ have demonstrated the potential to evoke protective immune responses. Although the two experiments were conducted independently, our findings revealed a more notable improvement in protection observed in both the lung and spleen of the HEHR/CIA09A subcutaneously boosted group when compared to the intramuscularly boosted group. Even, our previous vaccine, HSP90-E6, demonstrated increased efficacy when administered via subcutaneous boosting, significantly reducing both pulmonary inflammation and bacterial loads in the lung and spleen compared to the ESAT-6-only boosted group. This may be partly due to muscle tissue not being considered an optimal site for immunization, attributed to its low density of immune cells^65^. Additionally, targeting the same LNs through a homologous route for prime-boost vaccinations is suggested to provide greater boosted effects^36^. This prompted us to develop a tag-free version of HEHR and evaluate its efficacy by using the subcutaneous route first to boost BCG. Nevertheless, HEHR/CIA09A boosted vaccination, administered via both intramuscular and subcutaneous routes, demonstrated long-lasting superiority by significantly reducing pulmonary inflammation and bacterial burden against Mtb clinical strain M2, surpassing the efficacy of HSP90-E6 boosted vaccination. It is noteworthy that our vaccine candidate mediated pulmonary protection when vaccinated via the intramuscular route. Although we did not conduct a direct analysis of vaccine-specific CD4^+^ T-cell responses through flow cytometric analysis, elevated production of Th1/Th17-related cytokines was observed in the lungs from BCG-primed, intramuscularly HEHR/CIA09A-boosted mice. This may be partly because intramuscular injections can contribute to enhanced pulmonary protection and elicit mucosal immune responses. The intramuscular injection of vaccines, such as a replication-defective adenovirus vector and inactivated *Mycoplasma hyopneumoniae*, has been reported to induce mucosal immune responses in the lungs or intestines^66,67^. In addition, the M72/AS01_E_ vaccine demonstrated 49.7% efficacy in the final analysis, when administered via the intramuscular route, by preventing the progression to active pulmonary TB disease for at least 3 years^8^. Furthermore, our previous research, conducted in BCG-primed mice intramuscularly boosted with ID93/GLA-SE, demonstrated pulmonary protection against Mtb infections, consistent with observations in animal models vaccinated intramuscularly^42,68–70^. Given these insights, BCG-primed mice were intramuscularly boosted with HEHR/CIA09A, and as observed, this boosted regimen appeared to be effective in the lungs. On the other hand, boosting BCG with M72/AS01_E_ or H56:CAF01 via intramuscular route did not enhance protection along with Ag-specific CD4^+^ T-cell responses in Mtb-infected rhesus macaques^71^. Therefore, intramuscular booster vaccination with a tag-free version of HEHR should be further explored and considered in our future study, along with in-depth identification of correlates of protection. This will help optimize the appropriate vaccination route and enhance our understanding of the gap between vaccine-specific immune responses and protective efficacy.

It is widely acknowledged that TB vaccines designed to elicit Th1-cell-based immunity rely on a strong IFN-γ-mediated Th1 immune response^72,73^. However, IFN-γ response alone is not an optimal protective indicator^74^. Our previous data indicated that IFN-γ/IL-17A-producing multifunctional CD4^+^ T-cells exist, and their expansion contributes to enhanced Mtb protection^32^. In the current study, we also observed that ESAT-6-specific IFN-γ/IL-17A-producing multifunctional CD4^+^ T-cells were maintained at 12 weeks post-infection, and BCG-primed immune responses were notably elevated as evidenced by increased frequency of PPD-specific IFN-γ/IL-17A-producing multifunctional CD4^+^ T-cells in HEHR/CIA09A boosted mice. In addition, profiles of Th1/Th17-associated cytokine production upon stimulation of both Ags showed similar trends. Moreover, T-cells play a crucial role in infection control by differentiating into a protective effector state and adopting an effector phenotype to migrate to infected macrophage sites within the lung. Recently, in mouse models, it was reported that CXCR3^+^CD153^+^KLRG1^-^CD4^+^ T-cells, homing to the lung tissue, exhibit superior protection against Mtb in comparison to KLRG1^+^CD4^+^ T-cells associated with the vasculature, unable to access the lung tissue^37–39,75^. In line with this, we also observed an increased number of CXCR3^+^IFN-γ^+^IL-17A^+^ and KLRG^-^IFN-γ^+^IL-17A^+^ T-cells in the lung of HEHR/CIA09A boosted mice. However, as our limitation, we could not specifically demonstrate the phenotype of these T-cells based on discrimination of the lung compartments between lung parenchyma and vasculature through intravascular staining^37^. As ex vivo stimulation of Ags can affect the phenotype of T-cells during incubation, using tetramers for directly dissecting phenotypes of vaccine-specific T-cells should be also considered in our future study.

Accordingly, the protective potential of HEHR/CIA09A as BCG booster was consistently confirmed with the use of tag-free HEHR formulated in CIA09A by mediating long-term protection in terms of inflammation and bacterial burden compared to BCG-primed only. The reasons for tag-free antigens should be used in vaccines is that tagged proteins may potentially impact the safety and efficacy of Ags. In clinical trials, it is crucial to tightly control all factors and obtain predictable outcomes, making the use of tag-free Ags more desirable. Hence, tag-free Ags can streamline clinical trials by focusing on the antigen itself and eliminating unnecessary variables^40,76,77^. Korea is a mandatory BCG vaccination country, but still shows high TB incidence and mortality rates among OECD countries^1^, urgently necessitating the development of our own TB vaccine. To tackle this, our group has been trying to identify TB vaccine Ags and assess their efficacy against Mtb infections^32,33,46–50^. More recently, The Bill and Melinda Gates Foundation and Wellcome have committed $550 million to proceed with phase 3 clinical trials for an M72/AS01_E_ TB vaccine^78,79^. The M72 vaccine, as one of the most advanced TB vaccines, shares a common characteristic with other leading TB vaccines—ID93 and H56. They all belong to the category of multi-antigen, single-fusion protein vaccines, each uniquely formulated with its adjuvant. To meet this trend, tag-free Ags with our own adjuvants should be essentially required for developing our own TB vaccine in Korea.

Collectively, the multi-stage subunit vaccine HEHR, comprising four antigens, exhibits strong immunogenicity and remarkable long-lasting protective efficacy against Mtb clinical strain M2. The superior protection mediated by BCG-primed HEHR/CIA09A boosted vaccination was ensured by boosting BCG with a tag-free version of HEHR formulated in CIA09A. These findings hold significance for the design of effective TB vaccines and intend to progress toward future clinical trials.

## Methods

### Ethics statement

All animal studies were performed in accordance with Korean Food and Drug Administration (KFDA) guidelines. The experimental protocols used in this study were reviewed and approved by the Ethics Committee and Institutional Animal Care and Use Committee (Permit Number: 2020-0126) of the Laboratory Animal Research Center at Yonsei University College of Medicine (Seoul, Korea) and IACUC (202009A-CNU-132) of animal care at Chungnam National University (Daejeon, Korea).

### Animals

Six- to seven-week-old specific pathogen-free female C57BL/6 J mice were purchased from Japan SLC, Inc. (Shizuoka, Japan). Mice were maintained under barrier conditions in the ABSL-3 facility at the Yonsei University College of Medicine. All mice were housed in a constant temperature/humidity environment (24 ± 1 °C, 50 ± 5%) under light-controlled conditions (12 h light-dark cycle; 7 am on and 7 pm off) and fed a sterile commercial mouse diet with ad libitum access to water.

### Expression and purification of recombinant proteins

First, for the construction of fusion protein *HspX-RipA*, a two-step strategy combining the assembly PCR and overlap extension PCR processes was using Mtb H37Rv ATCC27294 genomic DNA as a template and the following primers: *HindIII-RipA* forward, 5′- TCCGTCGACAAGCTTGATCCACAGACGGACACC-3′, and *RipA* reverse, 5′-GGTGGTGGCCATGTACTCGATGTATCGGAC-3′, *HspX* forward, 5′-TACATCGAGTACATGGCCACCACCCTTCCC-3′, and *HspX-NotI* reverse, 5′-GCTCGAGTGCGGCCGCGTTGGTGGACCGGATCTG-3′. It was inserted into the pET22b vector. Second, for preparation of fusion protein *HSP90-ESAT6* DNA, the corresponding gene was amplified by PCR using recombinant plasmid pET22b containing *HSP90-ESAT6* cDNA as a template and the following primers: *NdeI-HSP90* forward, 5′-CATATGAACGCCCATGTCGAGCAGTTG-3′ and *ESAT6-HindIII* reverse, 5′-AAGCTTTGCGAACATCCCAGTGACGTT-3′. The PCR product of *HSP90-ESAT6* was digested with *Nde*I and *HindIII*, and inserted into pET22b+*HspX-RipA*, finally producing complete pET22b + *HSP90-ESAT6-HspX-RipA* (*HEHR*), and the resultant plasmids were sequenced. The recombinant protein was prepared as previously described^80^ and tag-free HEHR antigen was produced through the GenScript (Piscataway, NJ, USA). All the uncropped and unprocessed gel images are provided in the Supplementary Fig. 7.

### Preparation of mycobacteria strains

Mtb M2 strains were obtained from the strain collection of the International Tuberculosis Research Center (ITRC, Changwon, Gyeongsangnam-do, South Korea). BCG (Pasteur strain 1173P2) was kindly provided by Dr. Brosch from the Pasteur Institute (Paris, France). All mycobacteria used in this study were prepared as described previously^81^.

### Preparation of vaccine

Cationic liposomes were prepared from DOTAP and DMPC at a molar ratio of 1:1 using the thin-film method as previously described^82^. Briefly, a mixture of a chloroform solution containing DOTAP:DMPC = 1:1 (moral ratio) was dried in a round bottom flask using a rotary evaporator (IKA; Staufen, Germany) to form a lipid film. The lipid film was rehydrated with 10% (w/v) sucrose in 20 mM HEPS and 10% PBS (pH 7.4) solution and homogenized using a microfluidizer (Avestin; ON, Canada). The TLR4 agonist dLOS was prepared from an *E. coli* LPS-mutant strain as previously described^83^. Then, the adjuvant, CIA09A, was prepared by mixing cationic liposome (2 mg/ml of total lipid), 100 μg/ml of dLOS, and 20 μg/ml of QS-21 (Desert King International; CA, USA) and aliquoted in sealed glass vials, lyophilized and stored at 4 °C before use. A multiantigenic HSP90-ESAT-6-HspX-RipA (HEHR) vaccine was prepared by mixing HEHR antigen and a reconstituted form of lyophilized CIA09A. The vaccine was aliquoted into sealed glass vials, lyophilized, and stored at 4 °C. For characterization, lyophilized vaccines were rehydrated with deionized water and diluted tenfold with 10% (w/v) sucrose solution for analysis. The size, polydispersity index (PDI), and zeta-potential of vaccine particles were measured by dynamic light scattering (DLS) using Zetasizer Nano ZSP (Malvern Instruments, Worcestershire, UK). All measurements were performed in triplicate and analyzed at a detection angle of 173°, recording the temperature of 25 °C. Malvern Zetasizer DTS software (version 7.13) was used for analysis.

### Cell culture

Murine bone marrow-derived DCs and macrophages (BMDMs) were cultured and prepared as previously described^32^.

### Cell viability analysis

To investigate the effect of fusion proteins on cells, the cell death pattern of DCs was analyzed after treatment with HER, HEH, HERH, and HEHR (2 μg/ml). After 24 h of treatment, the harvested DCs were stained with FITC-Annexin V/PI (BD Biosciences) according to the manufacturer’s instructions. Staurosporine (STS) was treated as a positive control. Then, DC cell death was measured by using a CytoFLEX S flow cytometer (Beckman Coulter, Indianapolis, IN, USA) and analyzed using FlowJo version 10 software (TreeStar, Ashland, OR, USA).

### Measurement of cytokine production

Cytokines in supernatants of DC culture, DC co-cultured with T-cells, and single cells isolated from lungs of immunized mice were analyzed using commercial ELISA kits according to the manufacturer’s instructions. All ELISA kits were from Thermo Fisher Scientific, except for the IL-10 ELISA kit (Biolegend, San Diego, CA, USA).

### Analysis of surface molecule expression

After 24 h of treating DCs with fusion proteins (2 μg/ml), cells were stained with the following antibodies; anti-CD86 (PO3)-BV421 (BD Biosciences, San Jose, CA, USA), anti-MHC-II (M5/114.15.2)-APC-Cy7 (Biolegend).

### In vitro T-cell proliferation assay

About 1 μM CFSE-labeled CD4^+^ T-cells from splenocytes of OT-II mice were co-cultured with stimulated DCs (LPS; 100 ng/ml, fusion proteins; 2 μg/ml) pulsed with OVA_323-339_ peptide (Peptron, Daejeon, Korea) at a DC:T-cell ratio of 1:10 as previously described^84^.

### Assessment of bacterial growth inside macrophages

CD4^+^ T-cells from splenocytes of BCG-vaccinated mice were co-cultured with LPS- or fusion proteins-stimulated DCs at a ratio of 1:10 (DC:T-cell). Then isolated T-cells were added to Mtb-infected BMDMs and incubated for analyzing intracellular growth of bacteria as previously described^84^.

### Immunization and challenge protocol

BCG was subcutaneously vaccinated once with 1.0 × 10^6^ CFUs, then mice were immunized with subunit vaccine candidates (2 μg of antigen formulated in CIA09A) 10 weeks after BCG vaccination. These subunit vaccine candidates were given as a boost, intramuscularly or subcutaneously, three times at 3 weeks intervals. Four weeks after the final immunization, immunized mice were aerogenically challenged with the Mtb M2 strain as previously described^31^. Aerosol infection was performed using a Glas-Col aerosol apparatus (Terre Haute, IN, USA) adjusted to achieve an initial infectious dose of 200 CFUs. At 12 weeks post-challenge, mice from each group were euthanized in a euthanasia chamber following the gradual carbon dioxide (CO_2_) filling method from a compressed CO_2_ gas cylinder followed by KFDA guidelines for subsequent analysis.

### Intracellular cytokine staining

Single-cell suspensions of the lungs and spleens were prepared as previously described^30^. Briefly, single-cell suspensions (1 × 10^6^ cells) from infected or immunized mice were stimulated with ESAT6 (2 μg/ml) or HEHR (2 μg/ml) at 37 °C for 9 h in the presence of GolgiPlug and GolgiStop (BD Biosciences). For phenotypical analysis, single-cell suspensions were stained with the following antibodies; Thermo Fisher Scientific (Waltham, MA, USA): Live/Dead Fixable Viability Dye eFluorTM 780; Biolegend: anti-CD90.2 (53-2.1)-BV605, anti-CD44 (IM7)-PE-Cy7, anti-CXCR3 (CXCR3-173)-APC, anti-KLRG1 (2F1/KLRG1)-BV421; BD Biosciences: anti-CD4 (RM4-5)-PerCP-Cy5.5; Biolegend: anti-CD8a (53-6.7)-BV785, anti-CD62L (MEL-14)-Alexa700, anti-CD44 (IM7)-BV421. Then, cells were fixed, permeabilized, and stained intracellularly with following antibodies; BD Biosciences: anti-IFN-γ (XMG1.2)-PE, anti-IL-2 (JES6-5H4)-PE-Cy7, anti- TNF-α (MP6-XT22)-APC, anti-IL-17A (TC11-18H10)-Alexa488, anti-IL-17A (TC11-18H10)-APC-Cy7.

### Analysis of protective efficacy: bacterial enumeration and histopathology

After Mtb challenge, the lungs and spleens were homogenized. The number of viable bacteria was determined by plating serial dilutions of the organ homogenates onto Middlebrook 7H11 agar (Difco, Detroit, MI, USA) supplemented with 10% OADC (Difco) and amphotericin B (Sigma Aldrich, USA). Colonies were counted after 4 weeks of incubation at 37 °C. For histopathological analysis, the middle cross-section from the entire superior lobes of the right lung were stained with hematoxylin and eosin (H&E) and assessed for the severity of inflammation. The level of inflammation in the lungs was evaluated using by ImageJ (National Institutes of Health, USA) program, as previously described^68^.

### Statistical analysis

Data for all experiments are presented as the mean ± SD. For immunological analysis, the levels of significance for comparison between samples were determined by Tukey’s multiple comparison or unpaired *t*-test. For CFU and histopathology analysis, the Mann–Whitney rank test was used when comparing the differences between two different groups. For statistical analysis, GraphPad Prism version 8.00 for Windows was used (GraphPad Software, La Jolla California USA, www.graphpad.com). Differences having *p < 0.05, **p < 0.01, ***p < 0.001, or ****p < 0.0001 were considered statistically significant.

### Reporting summary

Further information on research design is available in the Nature Research Reporting Summary linked to this article.

### Supplementary information

Supplementary Information

Reporting Summary

## Data Availability

All data upon which conclusions are drawn are included in the manuscript or in the supplemental information file provided.
