# Supplementary material for: BCG-booster vaccination with HSP90-ESAT-6-HspX-RipA multivalent subunit vaccine confers durable protection against hypervirulent Mtb in mice
Source: NPJ Vaccines. 2024 Mar 8;9:55. doi: 10.1038/s41541-024-00847-7 (PMC10923817; doi:10.1038/s41541-024-00847-7)
Supplement: Supplementary file 1 — Supplementary Information [file 41541_2024_847_MOESM1_ESM.pdf]

BCG-booster vaccination with HSP90-ESAT-6-HspX-RipA multivalent-subunit vaccine confers durable protection against hypervirulent Mtb in mice

Kee Woong Kwon[1, 2†], Han-Gyu Choi[3†], Kwang Sung Kim[5], Shin Ae Park[5], Hwa-Jung Kim[3*], and Sung Jae Shin[1,4*]

[1]Department of Microbiology, Graduate School of Medical Science, Brain Korea 21 Project, Yonsei University College of Medicine, Seoul 03722, South Korea

[2]Department of Microbiology, College of Medicine, Gyeongsang National University, Jinju 52727, South Korea.

[3]Department of Microbiology, and Medical Science, College of Medicine, Chungnam National University, Daejeon 35015, South Korea

[4]Institute for Immunology and Immunological Disease, Yonsei University College of Medicine, Seoul 03722, South Korea

[5]R&D Center, EyeGene Inc., Goyang 10551, South Korea

[†]These authors contributed equally

***Address correspondence to:**

Sung Jae Shin, Department of Microbiology, Yonsei University College of Medicine, Seoul 03722, Republic of Korea. Phone: 82-2-2228-1813; Email: sjshin@yuhs.ac

Hwa-Jung Kim, M.D., Ph.D., Department of Microbiology, College of Medicine, Chungnam National University, 6 Munwha-Dong, Jung-Ku, Daejeon 301-747, South Korea; Tel.: +82-42-580-8242; Fax: +82-42-585-3686, E-mail: hjukim@cnu.ac.kr

Supplementary information

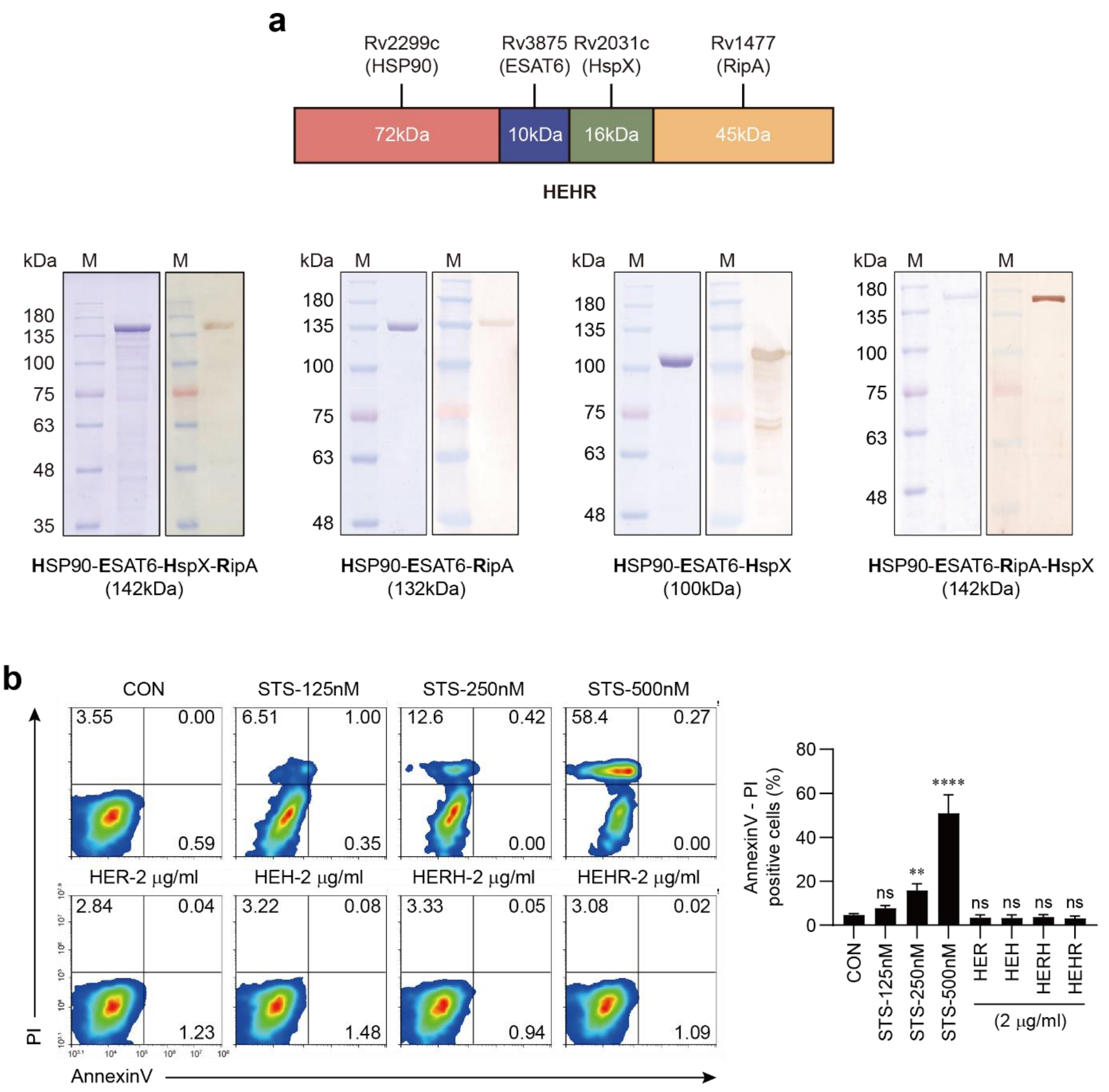

Supplementary Figure 1. Preparation and cytotoxicity of the recombinant fusion proteins. **a** Schematic diagram of the structure of HEHR. Purified recombinant HER, HEH, HERH, and HEHR protein was analyzed by SDS-PAGE with Coomassie blue staining and western blot analysis using anti-His antibodies. All the blots or gels were derived from the same experiment and were processed in parallel. **b** The cytotoxic effect of HER, HEH, HERH, and HEHR fusion proteins on DCs were analyzed by flow cytometry. HER, HEH, HERH, and HEHR fusion

proteins (2 μg/ml), and staurosporine (125 – 500 nM) was treated, and the cultures were harvested 24 h later. The DCs were stained with annexin V, and PI. The percentage of cells that are positive (annexin V- and PI-stained cells) in each quadrant is indicated. Graph shows mean ± SD. $^{**}P<0.01$ and $^{****}P<0.0001$: statistical significance of differences was determined compared to non-treated control.

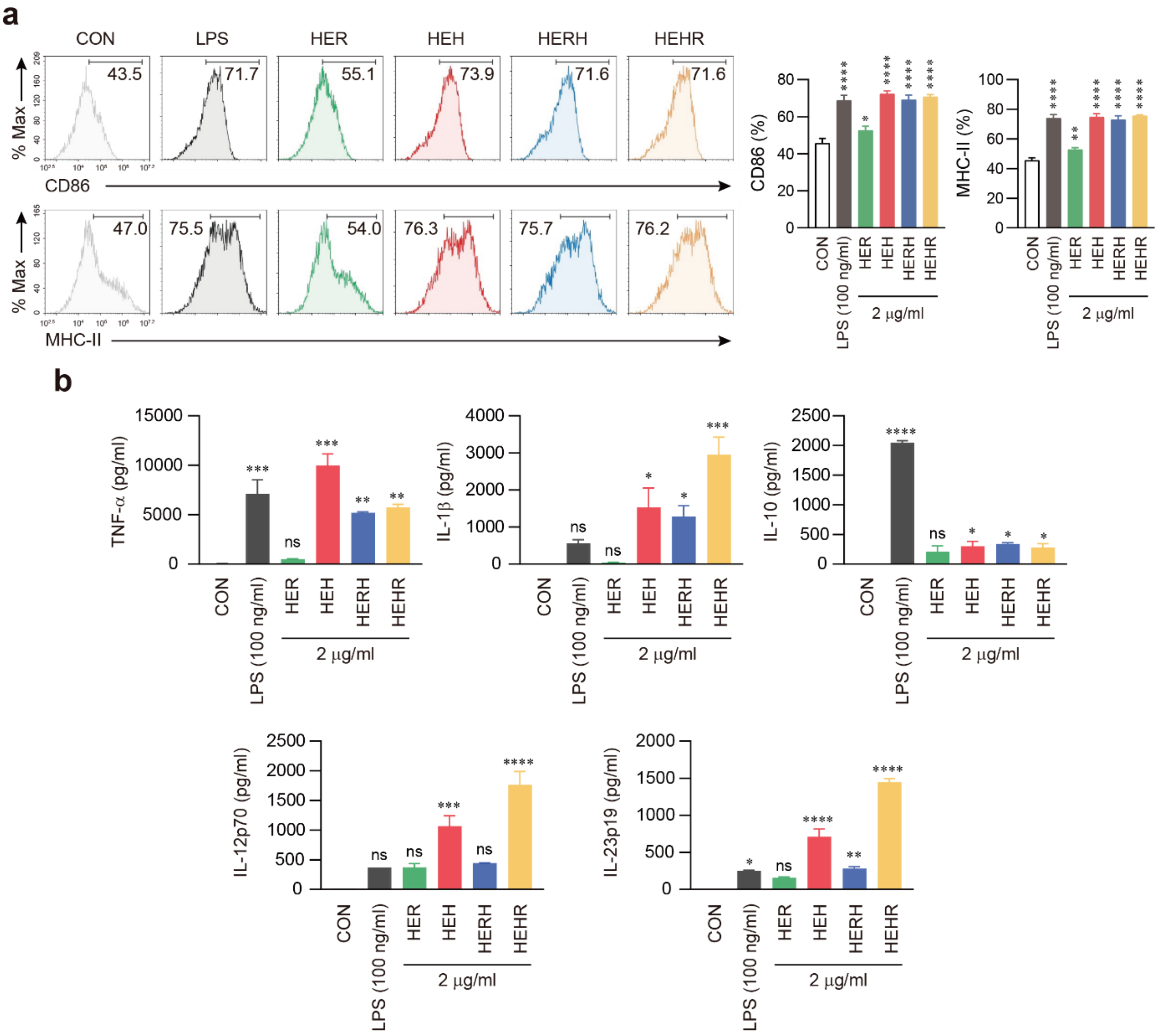

Supplementary Figure 2. **Fusion proteins induce DC maturation. a** Immature DCs (1×10^6 cells/ml) were cultured in the presence of HER, HEH, HERH, and HEHR fusion proteins (2 μg/ml), or 100 ng/ml LPS for 24 h and were analyzed for the expression of surface markers using flow cytometry. The cells were gated on $CD11c^+$. The DCs were stained with anti-CD86, or anti-MHC class II. The percentage of positive cells is shown in each histogram panel. **b** DCs were generated by stimulating immature DCs with HER, HEH, HERH, and HEHR fusion proteins (2 μg/ml), or 100 ng/ml LPS for 24 h. The quantities of TNF-α, IL-1β, IL-10, IL-12p70, and IL-23p19 in the culture supernatant were determined using an ELISA. The bar graphs show the mean ± SD (n = 3). **P*<0.05, ***P*<0.01, ****P*<0.001, and *****P*<0.0001 for

the treatments compared with untreated DCs; treatments that were not significantly different are indicated by ns.

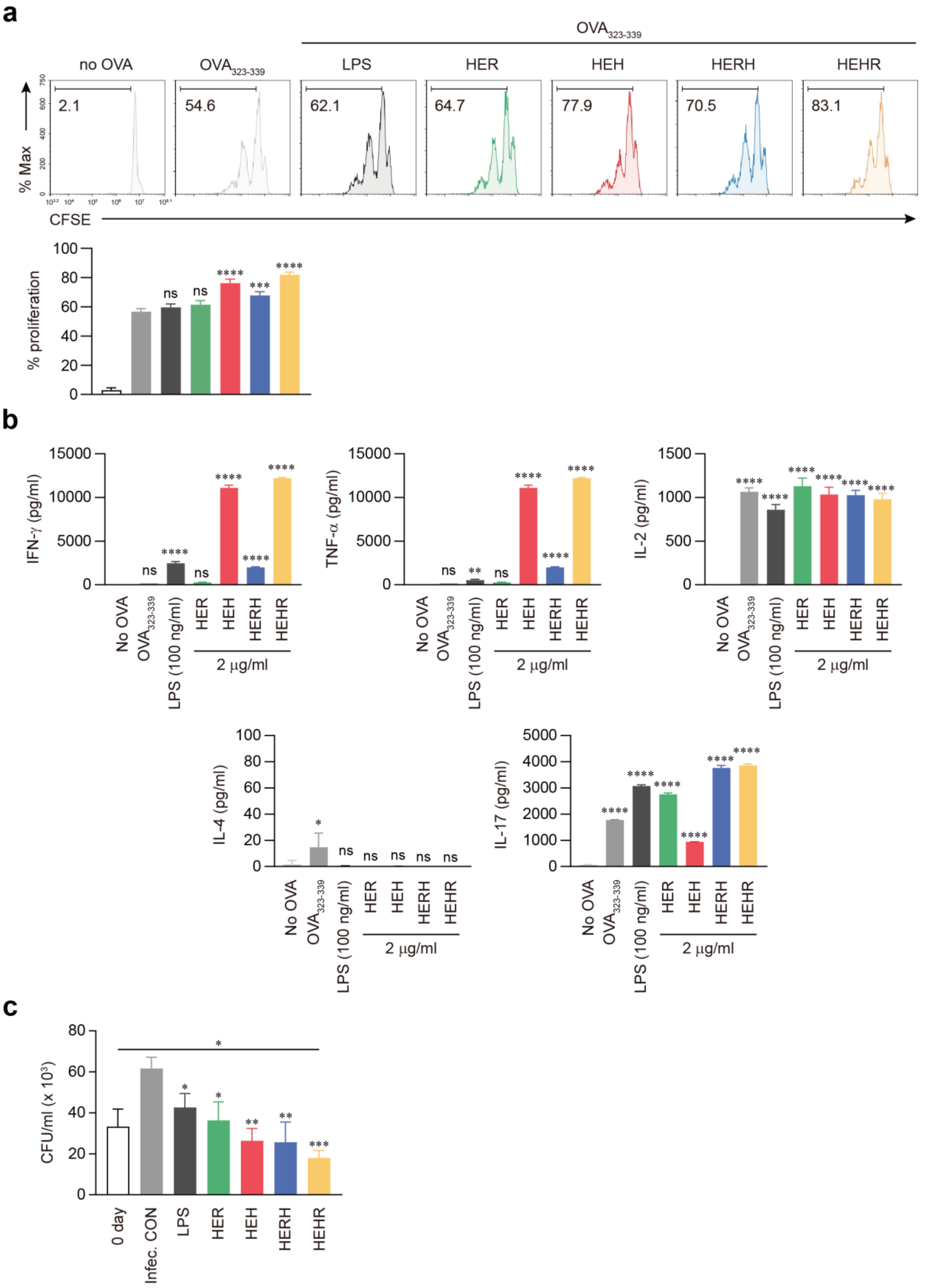

Supplementary Figure 3. T-cells activated by each fusion protein-treated DCs exhibited

high proliferation producing anti-mycobacterial Th1/Th17 cytokines. a CFSE-labeled OVA-specific $CD4^+$ T-cells were co-cultured with DCs treated with HER, HEH, HERH, and HEHR fusion proteins (2 μg/ml), or LPS (100 ng/ml). Prior to co-culture, untreated- or treated-DCs were pulsed with $OVA_{323-339}$ peptide (1 μg/ml). T-cells co-cultured with untreated DCs for 72 h co-culture were employed as control and the proliferation of T-cells were evaluated with flow cytometry. **b** After co-culture, cell supernatants were harvested and production of IFN-γ, TNF-α, IL-2, IL-4, and IL-17A was measured by ELISA. The bar graphs show the mean ± SD (n = 3). *n.s.*; not significant, $^*p < 0.05$, $^{**}P < 0.01$, $^{***}p < 0.001$, and $^{****}P < 0.0001$: statistical significance of differences was determined by comparing with appropriated control (T-cells co-cultured with $OVA_{323-339}$-pulsed untreated DCs). **c** Splenic $CD4^+$ T-cells isolated from BCG-vaccinated mice (4 weeks post vaccination) were co-cultured with untreated DCs, LPS- or each fusion protein-treated DCs for 72 h at a DC : T-cell ratio of 1 : 10. After co-culture, bacterial burdens inside bone-marrow derived macrophages was enumerated after co-culture with T-cells or without T-cells. The bar graphs show the mean ± SD (n = 3). *n.s.*; not significant, $^*P < 0.05$, $^{***}p < 0.001$, and $^{****}P < 0.0001$: statistical significance of differences was determined by comparing with T-cells co-cultured with untreated DCs or initial infection control.

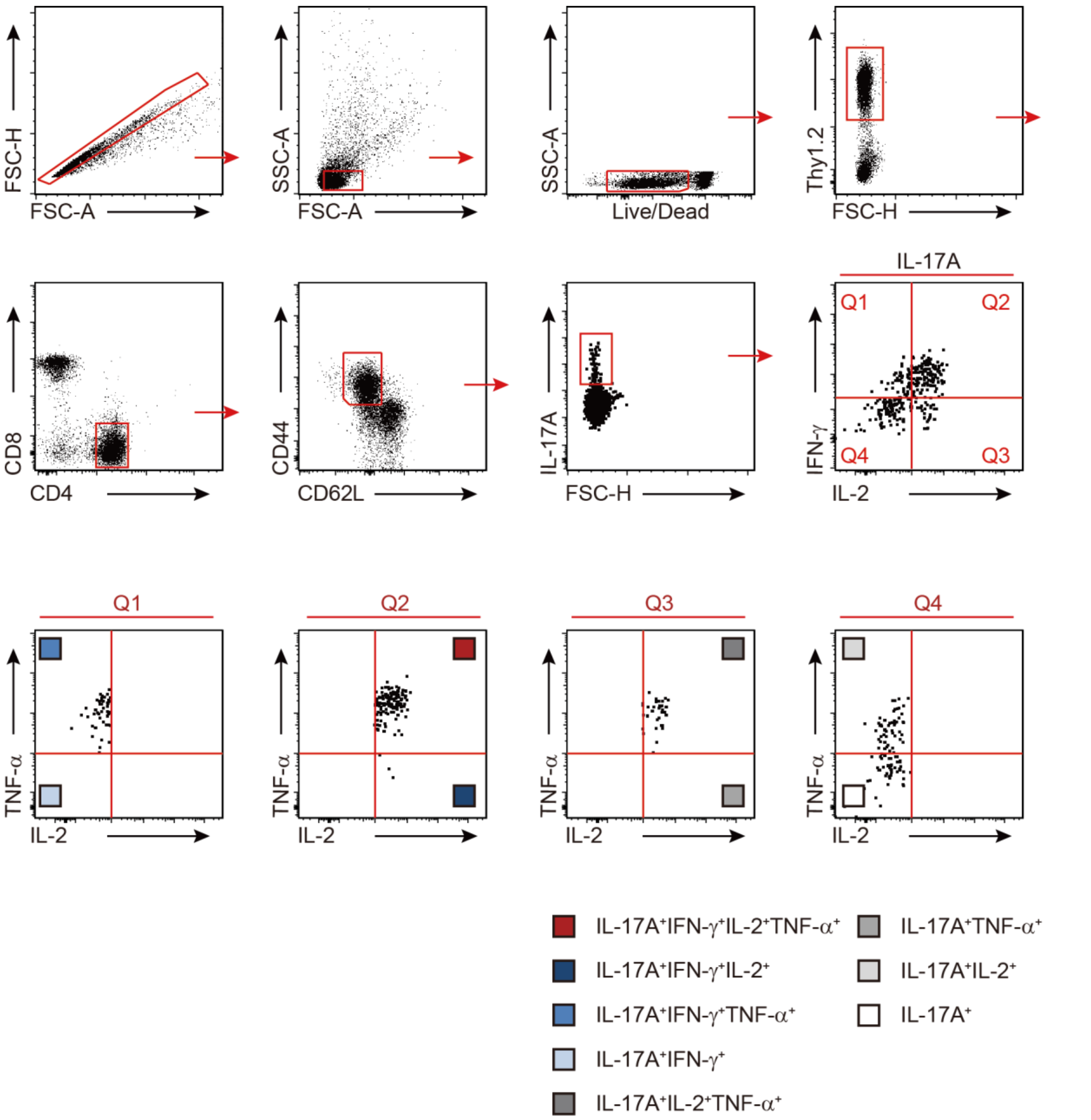

Supplementary Figure 4. Gating strategy of analyzing antigen-specific polyfunctional CD4$^+$ T-cells. All samples stained for surface and intracellular cytokines were gated based on forward scatter (FSC) and side scatter (SSC). T-cells were gated based on CD4 expression. Specific staining for intracellular cytokines is displayed using CD4$^+$ CD44$^+$ CD62L$^-$ T-cell gating.

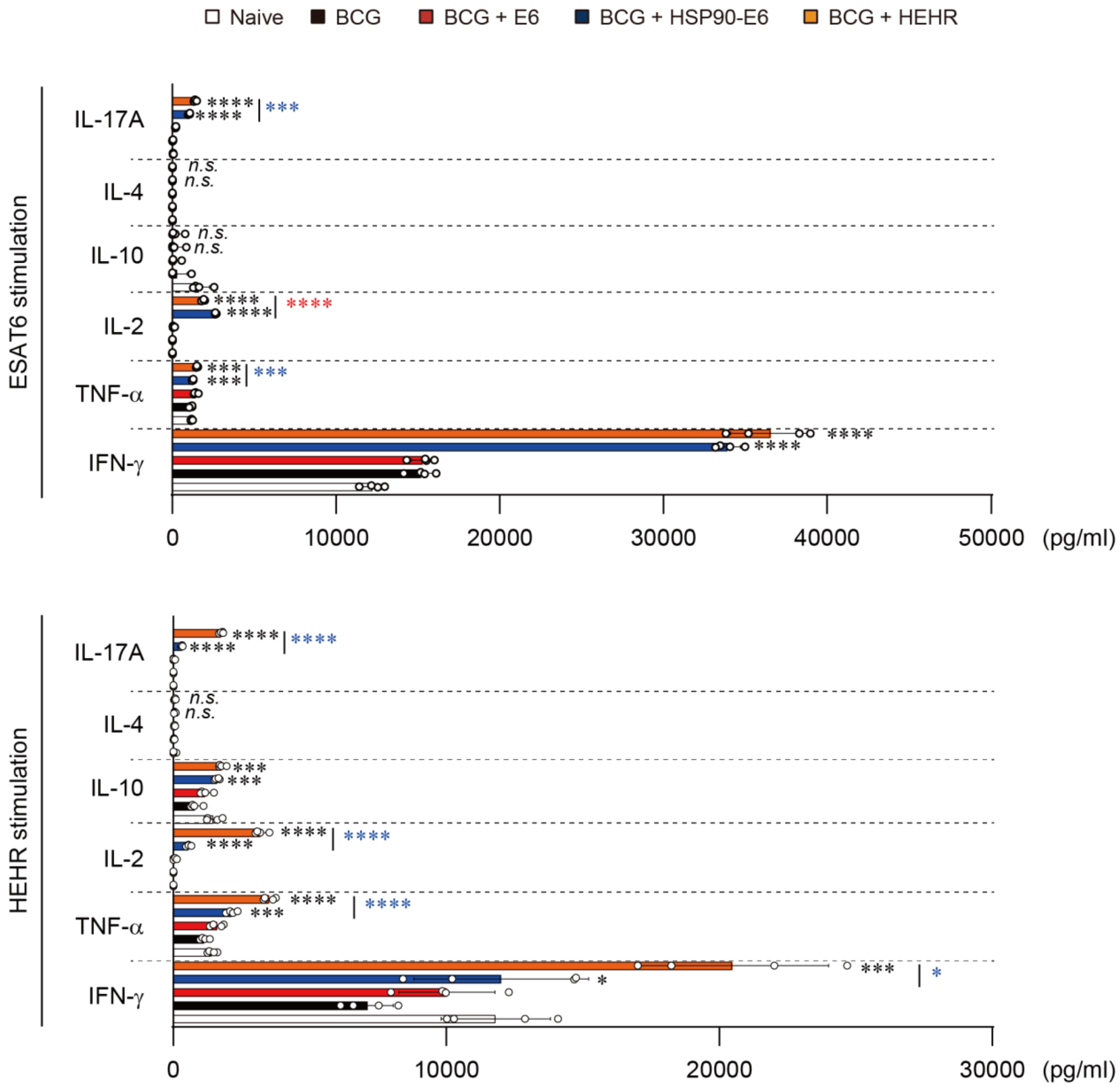

Supplementary Figure 5. Comparative cytokine production profiles in BCG-, BCG+E6-, BCG+HSP90-E6-, or BCG+HEHR-vaccinated mice upon Ag stimulation 4 weeks after final vaccination. Levels of IFN-γ, TNF-α, IL-2, IL-10, IL-4, and IL-17A secreted by lung cells from each fully immunized group in response to ESAT-6 (2 μg/ml; top) or HEHR (2 μg/ml; bottom) stimulation as detected by ELISA. Graph shows the mean ± SD. **p* < 0.05, ****p* < 0.001 and *****p* < 0.0001 compared to BCG-immunized mice. **p* < 0.05, ****p* < 0.001 and *****p* < 0.0001 between BCG+HSP90-E6- and BCG+HEHR-immunized mice.

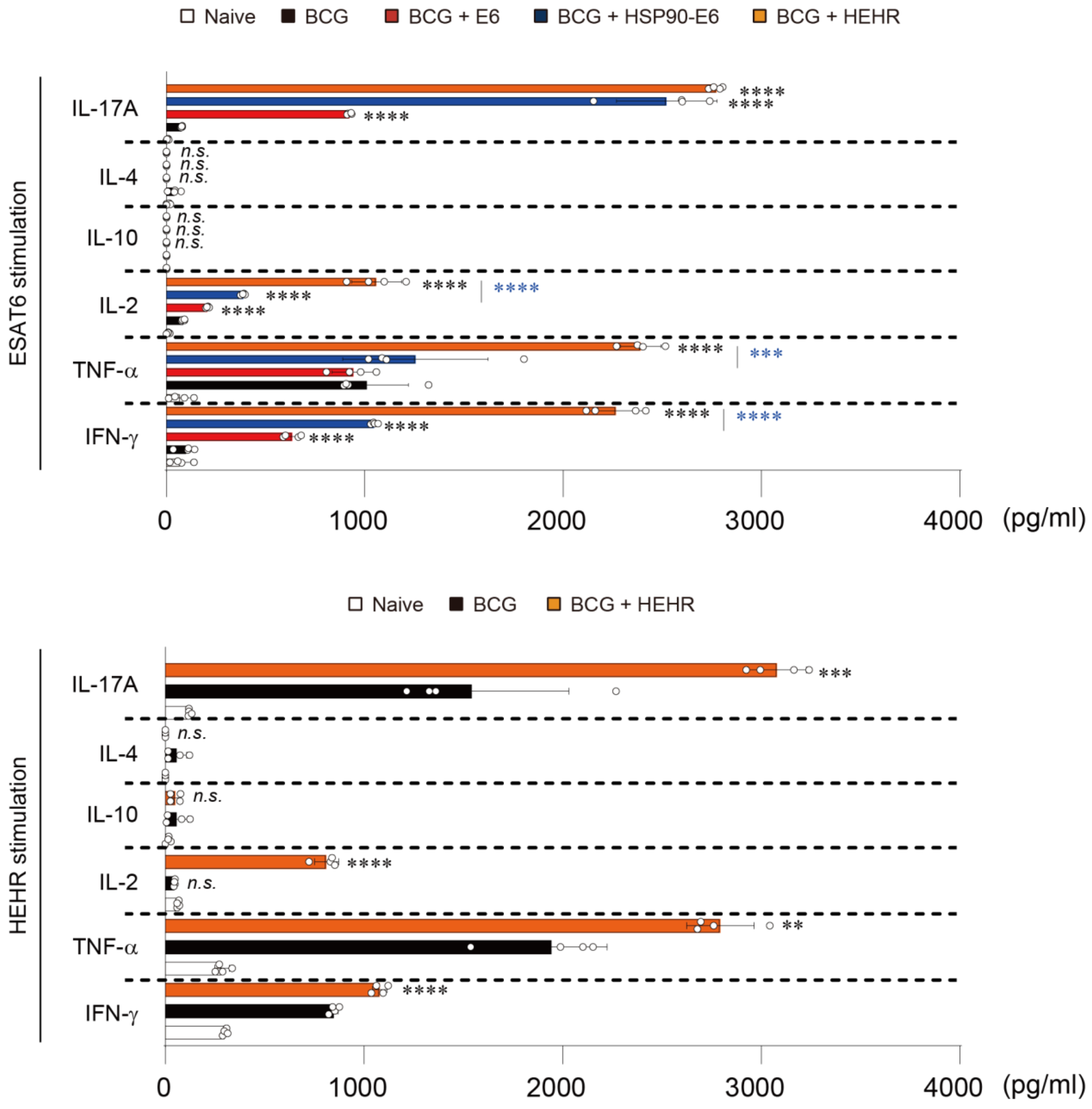

Supplementary Figure 6. Comparative cytokine production profiles in BCG primed intramuscularly Ag/CIA09A boosted mice upon Ag stimulation 4 weeks after final vaccination. Levels of IFN-γ, TNF-α, IL-2, IL-10, IL-4, and IL-17A secreted by lung cells from each fully immunized group in response to ESAT-6 (2 μg/ml; top) or HEHR (2 μg/ml; bottom) stimulation as detected by ELISA. Graph shows the mean ± SD. $**p < 0.01$, $***p < 0.001$ and $****p < 0.0001$ compared to BCG-immunized mice. $***p < 0.001$ and $****p < 0.0001$ between BCG+HSP90-E6- and BCG+HEHR-immunized mice.

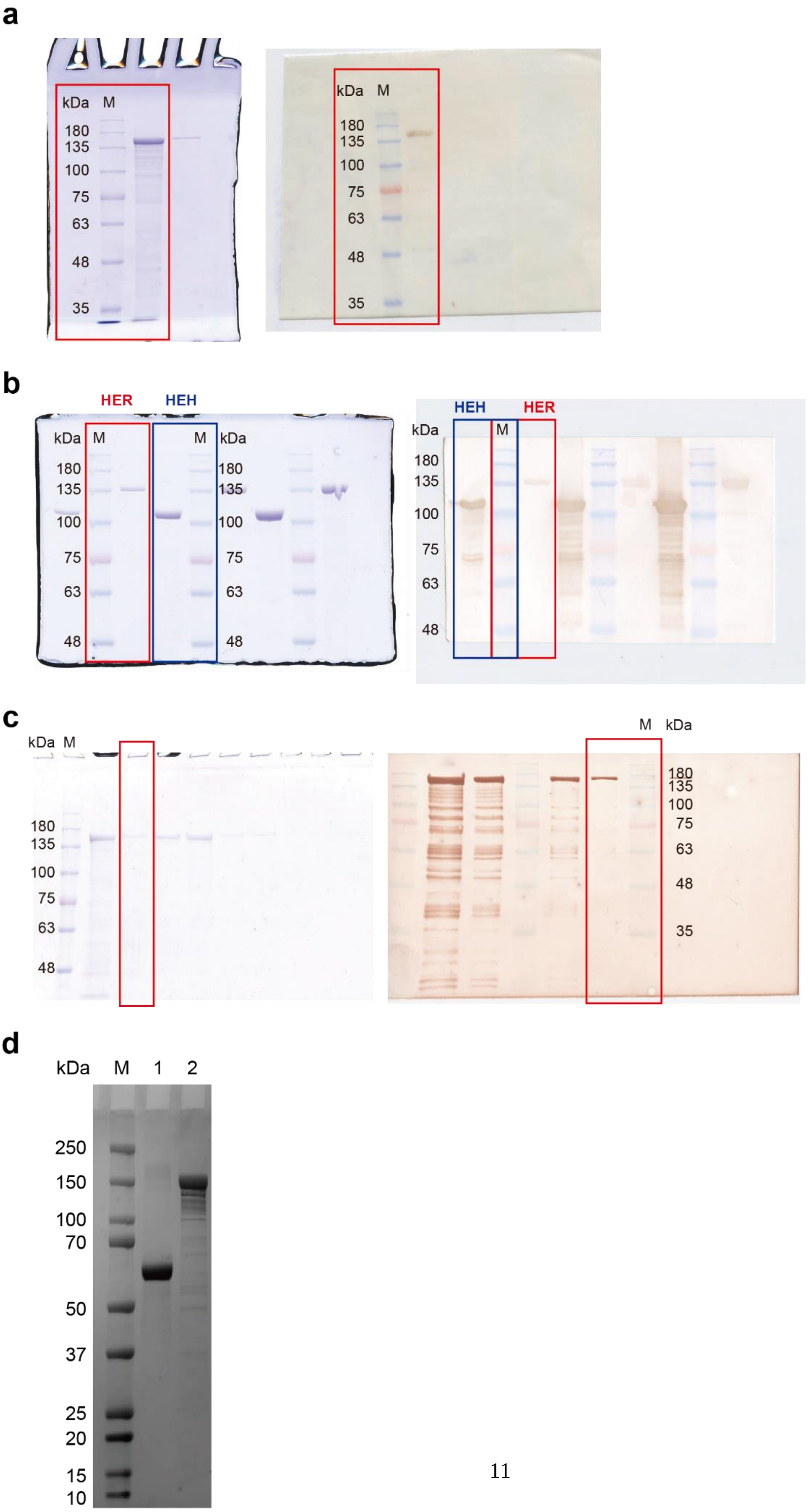
a
kDa M
180
135
100
75
63
48
35
kDa M
180
135
100
75
63
48
35
b
HER
HEH
kDa M
M kDa
180
135
100
75
63
48
HEH
HER
kDa
M
180
135
100
75
63
48
c
kDa M
180
135
100
75
63
48
M kDa
180
135
100
75
63
48
35
d
kDa M 1 2
250
150
100
70
50
37
25
20
15
10

Supplementary Figure 7. Images used to generate Supplementary Figure 1a and Figure 5a, showing SDS-PAGE gel and Western blot. a Uncropped and unprocessed images of HEHR corresponding to Supplementary Figure 1a. **b** Uncropped and unprocessed images of HER (red) and HEH (blue) corresponding to Supplementary Figure 1a. **c** Uncropped and unprocessed images of HERH corresponding to Supplementary Figure 1a. **d** Uncropped and unprocessed images of HEHR corresponding to Figure 5a.
